# Supplementary material for: Hypersensitivity reactions to multiple anti-tuberculosis drugs
Source: PLoS One. 2021 Feb 4;16(2):e0246291. doi: 10.1371/journal.pone.0246291 (PMC7861523; doi:10.1371/journal.pone.0246291)
Supplement: S1 Table — (DOCX) [file pone.0246291.s001.docx]

**S1 Table. Summary of enrolled subjects.**

| **No** | **Sex** | **Age** | **Exposure  to symptom, day** | **Initial symptoms** | **Symptom to OPT, day** | **Phenotype** | **Skin**  **prick test** | **Patch**  **test** | **Culprit drugs** | **Symptoms and signs of OPT** | **Type of DHR** | **Simultaneous/**  **sequential** |
| --- | --- | --- | --- | --- | --- | --- | --- | --- | --- | --- | --- | --- |
| **1** | F | 74 | 14 | Skin rash | 87 | MPE | NE | Neg | Ethambutol | Pruritus | delayed | – |
| **2** | M | 70 | 60 | Skin rash  Pruritus | 56 | MPE | NE | Neg | Ethambutol | Pruritus  Rash | delayed | – |
| **3** | F | 68 | 16 | Skin rash  Fever | 18 | MPE | NE | NE | Rifampin | Fever | delayed | – |
| **4** | F | 53 | 86 | Skin rash  Pruritus | 72 | MPE | NE | Neg | Ethambutol | Pruritus  Rash | delayed | – |
| **5** | F | 60 | 12 | Skin rash  Pruritus | 10 | MPE | NE | Neg | Ethambutol | Pruritus  Rash | delayed | – |
| **6** | M | 78 | 12 | Skin rash  Pruritus | 16 | MPE | NE | NE | Rifampin | Rash | delayed | – |
| **7** | F | 70 | 0 | Hypotension  Syncope  Fever | 112 | Anaphylaxis | Neg | NE | Rifampin | Urticarial  Angioedema | immediate | – |
| **8** | M | 52 | 12 | Skin rash  Fever  Eosinophilia | 17 | DRESS | NE | Neg | Rifampin | Pruritus  Rash | delayed | – |
| **9** | M | 55 | 2 | Skin rash  Fever | 25 | MPE | NE | NE | Rifampin | Pruritus | immediate | – |
| **10** | F | 40 | 11 | Skin rash  Pruritus | 47 | MPE | NE | Neg | Pyrazinamide | Pruritus | delayed | – |
| **11** | F | 25 | 7 | Skin rash  Pruritus | 27 | MPE | NE | NE | Rifampin | Pruritus  Rash | delayed | – |
| **12** | F | 61 | 4 | Skin rash  Pruritus | 84 | MPE | NE | NE | Ethambutol | Pruritus  Rash | delayed | – |
| **13** | M | 49 | 44 | Skin rash  Eosinophilia | 26 | MPE | NE | NE | Ethambutol | Pruritus  Rash | delayed | – |
| **14** | F | 31 | 30 | Skin rash  Pruritus | 38 | MPE | NE | NE | Rifampin | Pruritus  Rash | delayed | – |
| **15** | M | 71 | 133 | Skin rash  Eosinophilia | 77 | MPE | NE | NE | Rifampin | Pruritus  Rash | delayed | – |
| **16** | M | 75 | 35 | Skin rash  Pruritus | 28 | MPE | NE | NE | Ethambutol | Pruritus  Rash | delayed | – |
| **17** | F | 80 | 41 | Skin rash  Pruritus | 90 | MPE | NE | NE | Ethambutol | Pruritus  Rash | delayed | – |
| **18** | M | 81 | 86 | Skin rash | 73 | MPE | NE | Neg | Isoniazid | Pruritus  Rash | immediate | simultaneous |
|  |  |  |  |  |  |  |  |  | Pyrazinamide | Pruritus  Rash | delayed | simultaneous |
|  |  |  |  |  |  |  |  |  | Cycloserine | Pruritus  Rash | delayed | sequential |
| **19** | F | 74 | 7 | Urticaria  Pruritus | 28 | Urticaria | NE | NE | Rifampin | Vomiting  Urticaria  Dyspnea | immediate | simultaneous |
|  |  |  |  |  |  |  |  |  | Pyrazinamide | Pruritus  Rash | delayed | simultaneous |
| **20** | M | 69 | 6 | Skin rash  Fever | 69 | MPE | NE | NE | Isoniazid | Pruritus  Rash | delayed | simultaneous |
|  |  |  |  |  |  |  |  |  | Rifampin | Fever  Rash | delayed | simultaneous |
|  |  |  |  |  |  |  |  |  | Ethambutol | Fever  Rash | delayed | simultaneous |
|  |  |  |  |  |  |  |  |  | Pyrazinamide | Pruritus  Rash | delayed | simultaneous |
| **21** | F | 39 | 65 | Skin rash  Pruritus | 113 | MPE | NE | NE | Isoniazid | Pruritus  Rash | delayed | simultaneous |
|  |  |  |  |  |  |  |  |  | Rifampin | Pruritus  Rash | delayed | simultaneous |
|  |  |  |  |  |  |  |  |  | Ethambutol | Pruritus  Rash | delayed | simultaneous |
|  |  |  |  |  |  |  |  |  | Pyrazinamide | Pruritus  Rash | delayed | simultaneous |
| **22** | F | 45 | 11 | Skin rash  Fever | 115 | MPE | NE | NE | Ethambutol | Fever  Pruritus | delayed | simultaneous |
|  |  |  |  |  |  |  |  |  | Pyrazinamide | Fever  Pruritus | delayed | simultaneous |
| **23** | F | 85 | 82 | Skin rash  Fever | 75 | MPE | NE | NE | Rifampin | Pruritus  Rash | delayed | simultaneous |
|  |  |  |  |  |  |  |  |  | Ethambutol | Pruritus  Rash | delayed | simultaneous |
|  |  |  |  |  |  |  |  |  | Moxifloxacin | Pruritus  Rash | delayed | simultaneous |
| **24** | F | 44 | 11 | Skin rash  Fever  Eosinophilia | 24 | DRESS | NE | Neg | Isoniazid | Fever  Rash | delayed | simultaneous |
|  |  |  |  |  |  |  |  |  | Rifampin | Rash | delayed | simultaneous |
| **25** | F | 23 | 5 | Skin rash  Fever  Dyspnea | 59 | Anaphylaxis  MPE | Pos | NE | Rifampin | Fever  Rash | delayed | simultaneous |
|  |  |  |  |  |  |  |  |  | Pyrazinamide | Fever  Pruritus | delayed | simultaneous |
|  |  |  |  |  |  |  |  |  | Moxifloxacin | Flushing  Dyspnea  Hypotension | immediate | simultaneous |
| **26** | F | 36 | 1 | Skin rash  Eosinophilia | 20 | MPE | NE | NE | Rifampin | Pruritus  Rash | delayed | simultaneous |
|  |  |  |  |  |  |  |  |  | Ethambutol | Fever  Rash | delayed | simultaneous |
| **27** | F | 39 | 25 | Skin rash  Pruritus | 37 | MPE | Neg | Neg | Rifampin | Fever  Pruritus | delayed | simultaneous |
|  |  |  |  |  |  |  |  |  | Ethambutol | Fever | delayed | simultaneous |
| **28** | M | 18 | 12 | Skin rash  Eosinophilia | 38 | DRESS | NE | NE | Rifampin | Rash | delayed | simultaneous |
|  |  |  |  |  |  |  |  |  | Ethambutol | Rash | delayed | simultaneous |
|  |  |  |  |  |  |  |  |  | Moxifloxacin | Fever  Rash | delayed | sequential |

MPE, maculopapular exanthema; DRESS, drug reaction with eosinophilia and systemic symptoms; NE, not evaluated; OPT, oral provocation test; DHR, drug hypersensitivity reaction.
