# Supplementary material for: Hypersensitivity reactions to multiple anti-tuberculosis drugs
Source: PLoS One. 2021 Feb 4;16(2):e0246291. doi: 10.1371/journal.pone.0246291 (PMC7861523; doi:10.1371/journal.pone.0246291)
Supplement: Attachment — Submitted filename: COMMENTS TO AUTHOR.docx [file pone.0246291.s002.docx]

**PONE-D-20-16505
Hypersensitivity Reactions to Multiple Anti-tuberculosis Drugs**

Walter R. Taylor

Academic Editor

PLOS ONE

Dear Dr. Taylor,

Thank you for your letter dated August 26, 2020. We appreciate you and the reviewers for reviewing our manuscript entitled “Article Title: Hypersensitivity Reactions to Multiple Anti-tuberculosis Drugs” and offering helpful suggestions. We are submitting a revised manuscript that addresses the concerns that the reviewers raised. A detailed point-by-point response follows below.

We look forward to any additional comments or questions concerning this paper and hope that the revised manuscript is now acceptable for publication in PLOS ONE.

Sincerely,

Yong-Soo Kwon, on behalf of all the authors.

Associate professor

Department of Internal Medicine

Chonnam National University Hospital

Gwangju, Korea

E-mail: [yskwon@jnu.ac.kr](mailto:yskwon@jnu.ac.kr)

Reviewer #1: This manuscript addresses the issue of multiple drug hypersensitivity in the treatment of TBC. While there are published case reports, this seems to be the first retrospective chart analysis of a larger collective of patients.

**1. Major comment:**
**Q1. Methods**
Line 75 ff: Am I correct in understanding that you performed oral provocation tests in all patients with clinical suspicion of drug allergy without prior skin testing or performing other diagnostic tests? This would be not in line with current recommendations and guidelines on the management of drug allergy and could be highly dangerous in DRESS. Please specify. It seems, as if some patients at least did receive skin tests (discussion second but last paragraph). If so, please present the results.

**Response:** We agree with your comment that the oral provocation test is not recommended in patients with DRESS syndrome. However, since first-line anti-TB drugs are highly effective and few alternative effective anti-TB drugs are available, it is necessary to identify the culprit drug and use the remaining first-line anti-TB drugs. Therefore, we carefully performed the oral provocation tests using escalating doses as described in the manuscript. Of the 28 enrolled patients, two and eight patients underwent skin prick and patch tests, respectively, before the oral provocation test. One patient underwent both skin prick and patch tests before the oral provocation test. We have added this information in the Results section. Moreover, in this study, two patients with DRESS syndrome underwent patch tests before the oral provocation test; the patch test results were negative (Supplement table 1)

**Q2. Results:**
Q2-1) line 19/20 and table 1: it would be more interesting to discuss the time between start of treatment, first signs of hypersensitivity reaction, time between stop of treatment and oral challenge.

**Response:** Thank you for your comment. We have added the time interval between initiation of TB treatment and the occurrence of the first sign of DHR and the time interval between discontinuation of TB treatment and the oral provocation test in Table 1. We have also mentioned these findings in the Results section as follows: “There were no differences in the interval between initiation of TB treatment and the occurrence of the first sign of DHR and the interval between discontinuation of TB treatment and the oral provocation test between the two groups.”

Q2-2. Also: at the time of the challenges: where the patients treated with alternative/second line treatment for the TB? Any information on the inflammatory state (e.g.CRP, BSR, neutrophils, IL-6....) at the time

**Response:** Of the 28 patients, one patient underwent an oral provocation test while changing to second-line anti-TB drugs. The remaining 27 patients underwent oral provocation tests after discontinuing all anti-TB drugs. After identifying the causative drugs for DHRs, 19 and nine patients were administered modified anti-TB regimens during hospitalization and in outpatient clinics, respectively. We have added this in the Results section (Line 139). We have also added data on WBC count, neutrophil count, and CRP level, which serve as inflammatory markers on the oral provocation test day, in Table 1.

**Q3. table 1:**
Q3-1. calculating percentages in single cases of two collectives that overlap is misleading, e.g. in line "Previous treatment history": 1/28 patients in the whole collective and 1/17 patients in the subgroup result in different percentages, but in the end, this is only one patient. I suggest to omit the percentages from the small subgroups.

**Response:** We have omitted the percentages of the subgroups in Table 1 as per your recommendation.

Q3-2. skin rash: could you be more precise on the type and severity of clinical manifestation? macula-papular exanthema, AGEP, SDRIFE, FDE.......SJS/TEN

**Response:** We classified DHRs according to clinical manifestations, including MPE, anaphylaxis, DRESS syndrome, and SJS/TEN. We presented this information in Table 6.

Q3-3. table 2/3/4: please separate immediate type/anaphylaxis and delayed type reactions or alternatively, omit the few immediate cases from the table 4. One would not expect any elevation in liver parameters etc. in anaphylaxis.

**Response:** We agree with your comment on the unlikelihood of liver enzyme level elevation in patients with immediate-type hypersensitivity. To avoid complex tables presenting immediate- and delayed-type reactions with single and multiple DHRs, we have omitted two patients with immediate-type reactions, including one with single DHRs and one with multiple DHRs, in the comparison of AST and ALT levels in Table 4.

**Q4. Discussion**

Q4-1. line 15: what do you mean with “safely underwent the oral provocation tests”? Since you were able to confirm the clinical diagnosis of DHR by means of OPT those patients obviously reacted to the challenge, an at least unpleasant, if not to say potentially dangerous reaction that at least some of them could have been spared had you done a complete allergy workup beforehand.

**Response:** Thank you for your comment. We agree with your comment on the potential risk of OPT in patients with DHRs. Hence, we have deleted the sentence.

Q4-2. A separate table of the outcome of the diagnostic drug challenge as compared to the initial reaction would be useful with regard to the type and severity of the initial reaction, the type and severity of the reaction to the oral provocation and the further course of the treatment and potential further reactions etc. Did any of the patients with initial DRESS syndrome develop signs of systemic reaction upon oral provocation or did they “only” develop cutaneous signs?

**Response:** We have added a separate table for the clinical characteristics, oral provocation test results, and treatment outcomes according to the phenotypes of DHR.

**Table 6.** Clinical characteristics, oral provocation tests results, and treatment outcomes according to the clinical manifestations of DHRs

|  | MPE (n = 22) | Anaphylaxis (n = 3) | DRESS (n = 3) |
| --- | --- | --- | --- |
| Age, years (IQR) | 60.5 (39.7–74.2) | 70.0 (23.0–74.0) | 44.0 (18.0–52.0) |
| Female sex | 14 | 3 | 1 |
| Symptoms of DHR to OPT | Pruritus (n = 21)  Rash (n = 17)  Fever (n = 6)  Hypotension (n = 1)  Dyspnea (n = 1) | Pruritus (n = 2)  Rash (n = 2)  Fever (n = 1)  Urticaria (n = 2)  Angioedema (n = 1)  Hypotension (n = 1)  Dyspnea (n = 2) | Pruritus (n = 1)  Rash (n = 3)  Fever (n = 1) |
| Culprit drugs | INH (n = 3)  RIF (n = 11)  EMB (n = 14)  PZA (n = 5)  Mfx (n = 1) | RIF (n = 3)  Mfx (n = 1)  PZA (n = 2) | INH (n = 1)  RIF (n = 3)  EMB (n = 1) |
| Culprit drug and symptoms of further DHR after OPT | Cs (n = 1); pruritus and rash | - | Mfx (n = 1); fever and rash |
| Treatment success | 19 | 3 | 3 |
| Unfavorable outcomes | 3 | 0 | 0 |

Data are presented as number. MPE, maculopapular exanthema; DRESS, drug reaction with eosinophilia and systemic symptoms; IQR, interquartile range; OPT, oral provocation test; DHR, drug hypersensitivity reaction; INH, isoniazid; RIF, rifampin; EMB, ethambutol; PZA, pyrazinamide; Cs, cycloserine; Mfx, moxifloxacin

Q4-3. line 40 “There were discrepancies….” Could you further speculate on the reason for this discrepancy based on a comparison between your patient collective and the other ones published? Also, based on the mode of action of rifampicin and the other drugs in question?

**Response:** There could be differences in the frequency of DRESS syndrome according to different drugs used and patient age, sex, and race (Ann Allergy Asthma Immunol 123 (2019) 483-487). We have added this in the discussion section as follow: “possibly owing to different culprit drugs and patient characteristics, including age, sex, and race.”

Q4-4. line 53: cross reactivity between rifampicin and ethambutol? Is there evidence for that? If yes, based on what? The chemical structure and mode of action are quite different.

**Response:** To the best of our knowledge, no study has evaluated whether rifampicin and ethambutol have cross-reactivity. To clarify the meaning, we have modified the sentence as follows: “Further studies are needed to determine whether rifampicin and ethambutol influence each other in DHRs.”

Q4-5. line 58: please speculate on the reason for the reduced treatment success rate

**Response:** Thank you for your comment. We have added the following reason in the revised manuscript: “The discontinuation of effective anti-TB drugs, including rifampin, due to DHRs contributed to the low treatment success rate in this study.”

**2. Minor comments:**
**Q1. Introduction**

Q1-1. line 40 ff: please be more precise along the lines of the ICON-paper you have cited.. A DHR is not "similar" to drug allergy and DHR do usually not occur at any time or after "many" days

**Response:** Thank you for your comments. We made the following modification in the revised manuscript: “A drug hypersensitivity reaction (DHR) is an important adverse drug reaction (ADR) defined as “objectively reproducible symptoms or signs initiated by exposure to a defined stimulus at a dose tolerated by normal persons” by the World Health Organization (WHO). A drug reaction with demonstrated immunological mechanisms, either antibody or cell mediated, is referred to as drug allergy. DHRs can be classified as immediate or non-immediate DHRs depending on the onset time after drug exposure. Immediate DHRs typically occur within 1–6 h after drug exposure, whereas non-immediate DHRs commonly occur at any time after 1 h of drug administration” in the Introduction section (Line 41).

Q1-2. line 57: omit with

**Response:** Thank you for your comment. We have omitted it.

**Q2. Methods**
Q2-1. lines 79 ff: please specify the setting for immediate and delayed drug allergy: drug challenges in the hospital, at home, how long and how are the patients monitored

**Response:** All patients who underwent the oral provocation test were hospitalized, and the test was performed and monitored by an allergologist. We have added this in the Methods section (Line 78).

Q2-2. table 1: allergic disease: do you mean atopic disease? if yes, please change, if no, please specify
**Response:** Thank you for your comment. We have revised “allergic disease” to “atopic disease.”

Q3. Results (Comparisons of causative drugs and laboratory findings between single and multiple drug hypersensitivity groups, line 11 ff and table 4: this analysis is completely missing in the method section.

**Response:** Thank you for your comment. We have added the following in the Methods section: “Initial laboratory parameters at the start of anti-TB treatment and event laboratory parameters with the highest value when the patients had DHRs were collected. The laboratory parameters included white blood cell, lymphocyte, and eosinophil counts and percentages as well as aspartate aminotransferase (AST) and alanine aminotransferase (ALT) levels.” (Line 116)

Q4. Discussion in general, esp. last two paragraphs and figure 1: proofreading by a native speaker is needed, there are a number of grammatical errors.

**Response:** Thank you for your comment. Our manuscript has been proofread by a native English speaker.

Reviewer #2: This is a useful manuscript that documents outcomes of an oral challenge of patients who developed hypersensitivity reactions to first-line TB drugs. There are some improvements that can be made to this manuscript that would add significantly to its value in a field where there is limited data.

**Major Queries**
Q1. Line 92-95 – was DRESS the only reaction studied. If so then this should be clarified in the introduction. If not the criteria for diagnosing the others should be included

**Response:** Thank you for your comment. We have added the criteria for diagnosing anaphylaxis and SJS/TEN in the Methods section as follows (Line 102):

“SJS and TEN represent a spectrum of the most severe DHRs, wherein large areas of the epidermis become rapidly necrotic, resulting in skin detachment. SJS and TEN involve <10% and >30% of total body surface area, respectively.”

Q2. How as anaphylaxis diagnosed?

**Response:** We have added information regarding the diagnosis of anaphylaxis in the Methods section as follows (Line 100): “An anaphyaxis was defined as an acute onset of an illness involving the skin, mucosal tissue, or both in more than one body system after exposure to a trigger.”

Q3. Table 1 Are pruritus and fever always markers of hypersensitivity? Or are these part of a specific phenotype of ADR? It would be better if the authors classified/stratified the cases based on phenotype (i.e DRESS;anaphylaxis; sjs/ten; maculopapular etc.) The authors need to elaborate more on these.

**Response:** Thank you for your comment. We have modified Table 1 as per your comment. We have classified DHR in terms of the phenotype (MPE, anaphylaxis, DRESS syndrome, and SJS/TEN), which is presented in Table 1.

Q5. How were positive reactions to oral challenge determined? Were there any clinical features or only laboratory features? I suggest a table that includes every patient and the clinical features of a reaction, its timing etc. This will help others to identify the clinical features of this rechallenge reactions.

**Response:** We added the following details about the oral provocation test in the Methods section: “All patients were hospitalized for the oral provocation test when they had no symptoms related to DHRs; an allergologist and a trained nurse performed the test and monitored DHR-related symptoms and signs such as pruritus and skin rash. When the patient developed sign and symptoms of DHRs, the test was considered positive.” Further, we have added a table including all patients and their individual DHR-related characteristics, oral provocation test results, and DHR timing (Supplement 1).

Q6. It would be useful to detail the interval between initial ADR and challenge reaction for each patient and compare this to those who did not develop a challenge reaction. It is well established that the closer the challenge is performed to the index ADR the higher is the likelihood of a challenge reaction. (40 vs 28)

**Response:** Thank you for your comment. We have added the interval between the occur acne of the first sign of DHR and the oral provocation test in Table 1. However, we could not compare these in patients with positive and negative oral provocation test results. Of the 12 patients excluded from the study (Figure 1), only one patient underwent the oral provocation test; however, the patient was excluded as the final diagnosis was changed to NTM pulmonary disease. Of the remaining 11 patients excluded from the study, six and five patients underwent the skin prick test and the patch test, respectively.

Q7. Table 4. Does the initial event refer to the index drug reaction and which lab findings are reflected here. An average or the highest value during the event?

**Response:** Thank you for your comment. The initial laboratory parameters at the start of anti-TB treatment and event laboratory parameters with the highest value when the patients had the DHRs were collected. We added to this information in the Method section as follows: “We collected laboratory parameters related to anti-TB treatment initiation (initial values) and DHR occurrence (peak values). The laboratory parameters included white blood cell, lymphocyte, and eosinophil counts and percentages as well as aspartate aminotransferase (AST) and alanine aminotransferase (ALT) levels.”

Q8. Line 110 and table 4 – what were the unfavorable outcomes. Initially defined as including all but in the table they are separated.

**Response:** The definition of unfavorable outcome is stated in the Methods section as follows: “Treatment failure, death, and loss to follow-up were considered as unfavorable outcomes.” No patient in this study had treatment failure; therefore, we have omitted the number of patients with treatment failure in Table 5.

Q9. Lines 17 to 26 – the details of these reactions are useful addition to existing literature and even if added as supplementary material.
**Response:** Thank you for your valuable comment. Unfortunately, we could not add it, because we could not find any reference to sequential multiple DHR associated with anti-TB drugs.

**Minor Comments**
Q1. Line 49 – loss not lost

**Response:** We have changed the word as per your comment.

Q2. Line 52 – “Previously, we reported that peripheral eosinophilia during anti-TB therapy was common (17.8%) and that cutaneous ADRs were common in these patients” ADR is common in all patients or those with eosinophilia?

**Response:** Thank you for your comment. We made the following modification in the revised manuscript: “In our previous study, peripheral eosinophilia during anti-TB therapy was common (17.8%) and cutaneous ADRs were common in these patients with eosinophilia.”

Q3. Line 74 - anti-tuberculous change to anti-TB for consistency.

**Response:** Thank you for your comment. We have changed the word as per your comment.

Q4. Line 76 – “starting with the drug with the lowest probability of hypersensitivity,” how was this established? Please provide reference

**Response:** Thank you for your comment. The revised manuscript has been modified to clarify the meaning. “The oral provocation test was performed in the order of pyrazinamide, isoniazid, ethambutol, and rifampin. However, according to the judgment of the clinician, the order of anti-TB drugs for oral provocation test may be changed in the order of drugs with the lowest probability of hypersensitivity.”

Q5. Line 77 – was there any rationale/references for the protocol used? Pls elaborate

**Response:** The protocol used in this study was modified from the one we previously used in our hospital (Kim et al. Allergy Asthma Immunol Res. 2014 March;6(2):183-185). We have cited the reference in the revised manuscript.

Q6. Line 88 – “A simultaneous multiple DHR was the simultaneous occurrence of drug hypersensitivity to different administered drugs” is this the same definition as used by Gex-Collet et al?

**Response:** We have modified the manuscript as follows: “Simultaneous and sequential multiple DHRs were defined as the occurrence of hypersensitivity to different administered drugs simultaneously and at time intervals, respectively.”
Q7. Line 95 – meant to read “exclusion of other potential causes”

**Response:** We have corrected the word as per your comment.

Q8. Line 97-100 is a repetition

**Response:** We have omitted the sentence.

Q9) Line 5 – should read flowchart

A9. We have revised “flowcahrt” to “flowchart.”
