# Supplementary material for: Hypersensitivity reactions to multiple anti-tuberculosis drugs
Source: PLoS One. 2021 Feb 4;16(2):e0246291. doi: 10.1371/journal.pone.0246291 (PMC7861523; doi:10.1371/journal.pone.0246291)
Supplement: Attachment — Submitted filename: Response to comments of editor.docx [file pone.0246291.s003.docx]

**PONE-D-20-16505
Hypersensitivity Reactions to Multiple Anti-tuberculosis Drugs**

Walter R. Taylor

Academic Editor

PLOS ONE

Dear Dr. Taylor,

Thank you for your letter dated August 26, 2020. We appreciate you for reviewing our manuscript entitled “Article Title: Hypersensitivity Reactions to Multiple Anti-tuberculosis Drugs” and offering helpful suggestions. We are submitting a revised manuscript that addresses the concerns that the reviewers raised. A detailed point-by-point response follows below.

We look forward to any additional comments or questions concerning this paper and hope that the revised manuscript is now acceptable for publication in PLOS ONE.

Sincerely,

Yong-Soo Kwon, on behalf of all the authors.

Associate professor

Department of Internal Medicine

Chonnam National University Hospital

Gwangju, Korea

E-mail: [yskwon@jnu.ac.kr](mailto:yskwon@jnu.ac.kr)

1. Please give the reader an idea of the risk of developing an adverse drug reaction when on standard anti TB treatment reported by others.

A) Thank you for your comment. We added the risk of developing an adverse drug reactions in the Introduction section as you suggested: “However, adverse drug reactions (ADRs) to anti-TB drugs can cause significant morbidity, leading the discontinuation of these effective anti-TB drugs and worse outcomes. The risk of developing an ADR to anti-TB drugs has been variously reported from 8 to 85% depending on the population the studies enrolled and the severity of ADRs the studies evaluated”

2. Do you know how many patients were treated for TB during the time of your retrospective study? That would also give us an idea of risk.

A) Thank you for your comment. As described in Figure 1, a total of 2,347 patients received anti-TB treatment during the study period. We added it in the Results section.

3. In your previous study, you reported eosinophilia in patients with TB drug reactions. More detail is needed like the rash morphologies and the eosinophil counts.

A) Thank you for your comment. Since this is a retrospective study, we could not evaluate the rash morphologies, but we described the eosinophil counts related to anti-TB treatment initiation (initial values) and DHR occurrence (peak values) in Table 4.

4. Please add a few simple lines to explain to general physicians what a sequential and simultaneous drug reaction are.

A) Thank you for your comment. We added the additional explanation for sequential and simultaneous DHR in the Discussion section as “Multiple DHR is a hypersensitivity reaction to two or more chemically distinct drugs. It was classified two forms based on simultaneous and sequential sensitization of drugs leading to multiple DHRs; a simultaneous form is multiple sensitizations at the same time, and a sequential form is multiple sensitization in a temporal sequence.”.

5. There is a small error regarding the Wilcoxon Signed Rank test - it is used to assess paired (within group) differences.

A) Thank you for your valuable comment. We performed the Wilcoxon Signed Rank test to determine whether the peak lab was significantly elevated than the baseline lab (within group, paired test). We changed the description in the statistical analysis as “The Wilcoxon signed rank test was used to compare initial and peak laboratory variables within groups”

6. Please tell the reader how you made a successful diagnosis of a positive rechallenged test. Table 6 would be improved by detailing the key symptoms and signs of the initial drug reaction and what happened when that individual was rechallenged. Therefore, you need to list every patient. Rank them by initial reaction starting with MPE. Please delete treatment outcomes as this has already been covered in Table 5.

A) Thank you for your comment. We already described how can do the oral provocation test and when it was considered positive in the Method section. “All patients were hospitalized for the oral provocation test when they had no symptoms related to DHRs; an allergologist and a trained nurse performed the test and monitored DHR-related symptoms and signs such as pruritus and skin rash. When the patient developed sign and symptoms of DHRs, the test was considered positive.”

We added the initial symptoms of DHRs in table 6 and modified supplement table for describing symptoms and signs of every patient. Since the complexity of table, we listed symptoms and sings of every patient about of the initial drug reaction and what happened when that individual was rechallenged only in the supplement table.

7. Table 4. We need to see the significant p values within groups as well. As you seem to have the white cell data, a graph would be very nice showing the changes in total and white cell count differentials over time to cover also the oral provocation test (this is optional).

A) Thank you for your comments. We added the significant p values in comparisons between initial and peak values within groups in Table 4. We did not add a graph because the WBC was not significantly different between groups and between initial and peak values within groups, and it has already been covered in table 4.

8. It is worth reminding clinicians in the Discussion that drug reactions can occurs months after starting treatment and referral to a specialist unit for drug rechallenged should be made quickly.

A) Thank you for your comment. We added the additional comment to the Discussion section as your suggestion. “Clinicians should keep in mind that DHR can occur several months after the start of anti-TB treatment, and consider referral to a specialized unit to identify culprit drugs quickly.”.
